# 1,3-Difluoro­benzene

**DOI:** 10.1107/S1600536809038987

**Published:** 2009-10-07

**Authors:** Michael T. Kirchner, Dieter Bläser, Roland Boese, Tejender S. Thakur, Gautam R. Desiraju

**Affiliations:** a Institut für Anorganische Chemie der Universität, 45117 Essen, Germany; b Indian Institute of Science, Bangalore 560 012, India

## Abstract

The weak electrostatic and dispersive forces between C(δ+)—F(δ−) and H(δ+)—C(δ−) are at the borderline of the hydrogen-bond phenomenon and are poorly directional and further deformed in the presence of other dominant inter­actions, *e.g.* C—H⋯π. The title compound, C_6_H_4_F_2_, *Z*′ = 2, forms one-dimensional tapes along two homodromic C—H⋯F hydrogen bonds. The one-dimensional tapes are connected into corrugated two-dimensional sheets by further bi- or trifrucated C—H⋯F hydrogen bonds. Packing in the third dimension is controlled by C—H⋯π inter­actions.

## Related literature

For C—H⋯F inter­actions, see: Althoff *et al.* (2006 ▶); Bats *et al.* (2000 ▶); Choudhury *et al.* (2004 ▶); D’Oria & Novoa (2008 ▶); Dunitz & Taylor (1997 ▶); Howard *et al.* (1996 ▶); Müller *et al.* (2007 ▶); O’Hagan (2008 ▶); Reichenbacher *et al.* (2005 ▶); Weiss *et al.* (1997 ▶). For the crystal structures of polyfluorinated benzenes, see: Thalladi *et al.* (1998 ▶). For crystallization techniques, see: Boese & Nussbaumer (1994 ▶).
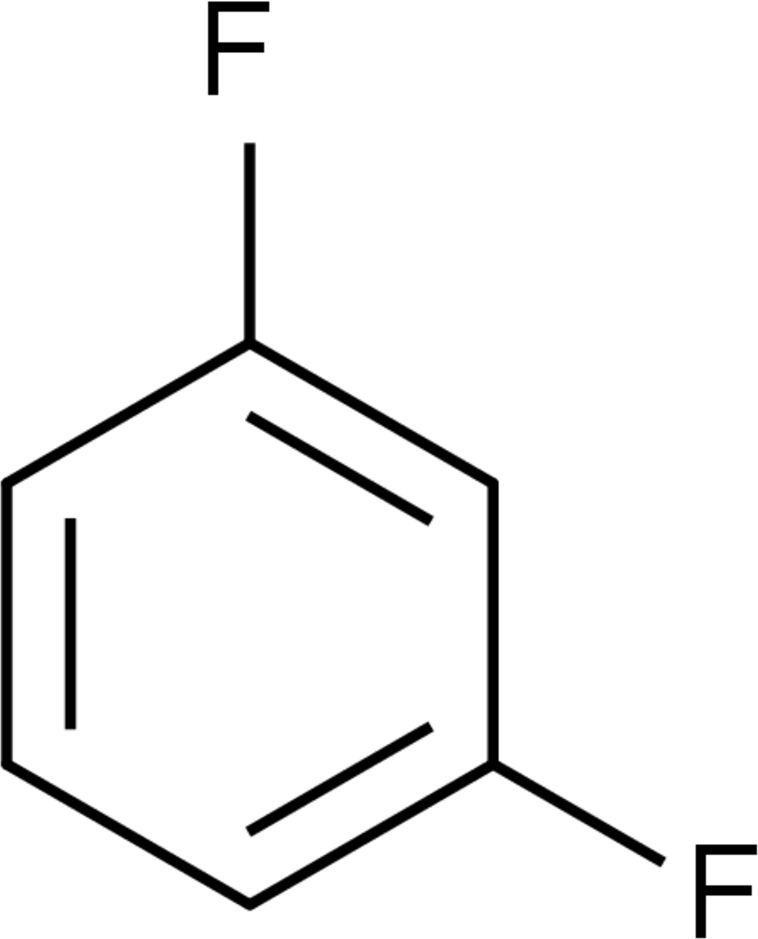


## Experimental

### 

#### Crystal data

C_6_H_4_F_2_
*M* *_r_* = 114.09
Monoclinic, 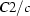

*a* = 24.6618 (13) Å
*b* = 12.2849 (5) Å
*c* = 7.2336 (4) Å
β = 106.842 (3)°
*V* = 2097.55 (18) Å^3^
*Z* = 16
Mo *K*α radiation
μ = 0.13 mm^−1^
*T* = 153 K
0.30 × 0.30 × 0.30 mm

#### Data collection

Bruker SMART APEXII area-detector diffractometer
Absorption correction: multi-scan (*SADABS*; Bruker, 2004 ▶) *T*_min_ = 0.876, *T*_max_ = 0.961
7831 measured reflections
2099 independent reflections
1578 reflections with *I* > 2σ(*I*)
*R*_int_ = 0.020

#### Refinement

*R*[*F* ^2^ > 2σ(*F* ^2^)] = 0.032
*wR*(*F* ^2^) = 0.100
*S* = 1.01
2099 reflections
146 parameters
H-atom parameters not refined
Δρ_max_ = 0.19 e Å^−3^
Δρ_min_ = −0.13 e Å^−3^

### 

Data collection: *APEX2* (Bruker, 2008 ▶); cell refinement: *SAINT* (Bruker, 2008 ▶); data reduction: *SAINT*; program(s) used to solve structure: *SHELXTL* (Sheldrick, 2008 ▶); program(s) used to refine structure: *SHELXTL*; molecular graphics: *Mercury* (Macrae *et al.*, 2008 ▶) and *GIMP* (The GIMP team, 2008 ▶); software used to prepare material for publication: *publCIF* (Westrip, 2009 ▶).

## Supplementary Material

Crystal structure: contains datablocks I, glonal. DOI: 10.1107/S1600536809038987/ci2886sup1.cif

Structure factors: contains datablocks I. DOI: 10.1107/S1600536809038987/ci2886Isup2.hkl

Additional supplementary materials:  crystallographic information; 3D view; checkCIF report

## Figures and Tables

**Table 1:** Hydrogen-bond geometry (Å, °)

*D*—H⋯*A*	*D*—H	H⋯*A*	*D*⋯*A*	*D*—H⋯*A*
C2—H2⋯F12^i^	0.96	2.72	3.3750 (14)	126
C4—H4⋯F2^ii^	0.96	2.76	3.5386 (16)	139
C5—H5⋯F11^iii^	0.95	2.71	3.2948 (16)	121
C6—H6⋯F11^iii^	0.96	2.66	3.2644 (15)	121
C6—H6⋯F1^iv^	0.96	2.82	3.5789 (17)	137
C12—H12⋯F1^v^	0.96	2.70	3.3919 (14)	130
C14—H14⋯F2^vi^	0.96	2.72	3.3442 (16)	123
C14—H14⋯F12^vii^	0.96	2.73	3.5075 (18)	138
C15—H15⋯F2^vi^	0.96	2.81	3.3995 (17)	120
C16—H16⋯F11^viii^	0.96	2.75	3.5591 (16)	142
C2—H2⋯*Cg*2^ix^	0.96	2.96	3.6653 (13)	131
C12—H12⋯*Cg*2^v^	0.96	2.99	3.6547 (13)	127
C5—H5⋯*Cg*1^x^	0.95	2.83	3.5153 (12)	130
C15—H15⋯*Cg*1	0.96	2.87	3.5283 (13)	127

Symmetry codes: (i) 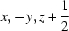; (ii) 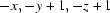; (iii) 
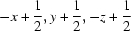
; (iv) 
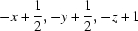
; (v) 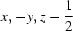; (vi) 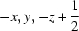; (vii) 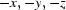; (viii) 
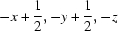
; (ix) 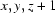; (x) 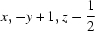. *Cg*1 and *Cg*2 are the centroids of the C1–C6 and C11–C16 rings, respectively.

**Fig. 1.**
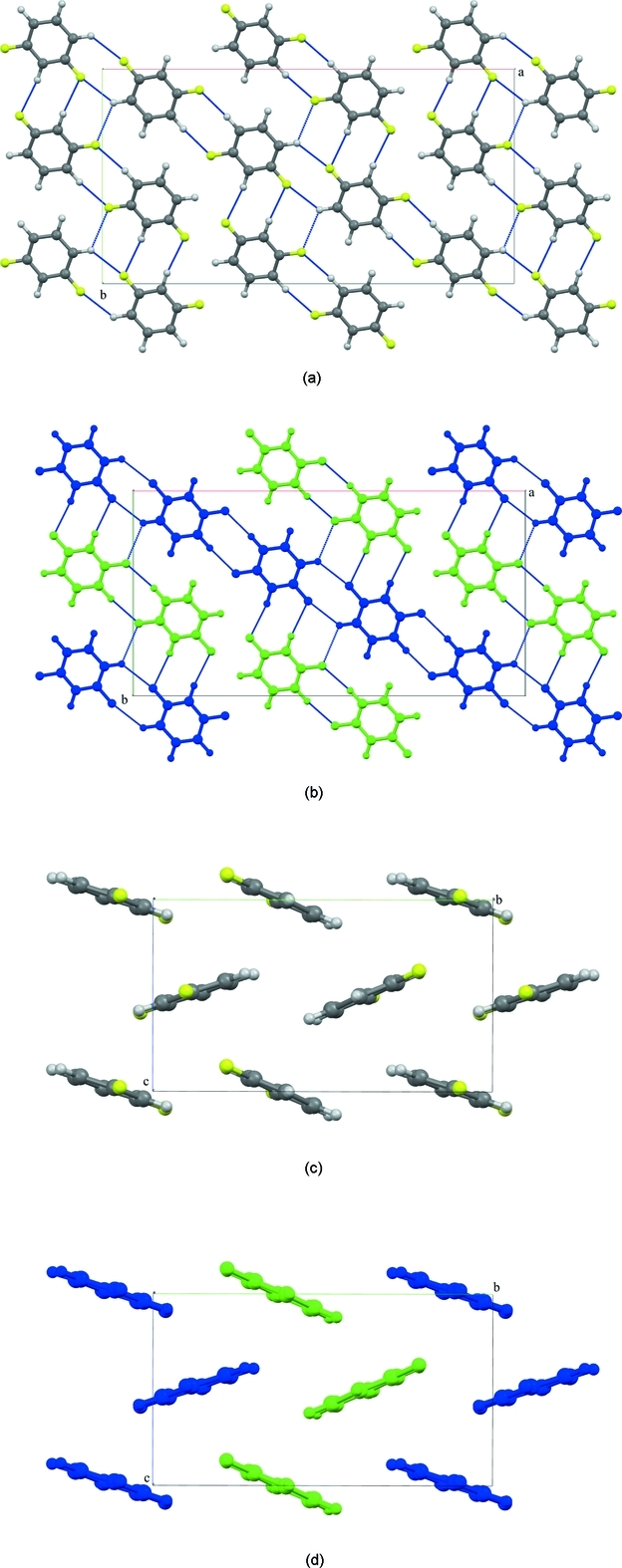
Crystal structure of 1,3-difluorobenzene: (a) two-dimensional network of C—H···F—C interactions viewed along the c axis, (b) with independent molecules coloured blue and green, (c) Herringbone arrangement of molecules viewed along the a axis and (d) coloured as before.

**Fig. 2.**
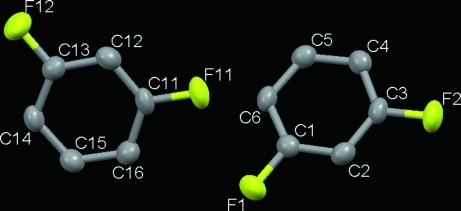
Displacement ellipsoid plot of 1,3-difluorobenzene.

## supplementary crystallographic information

### Comment

Despite the high electronegativity difference between carbon and fluorine, the C—F bond acts as a poor hydrogen bond acceptor due to the hardness of the F-atom (Dunitz & Taylor, 1997; O'Hagan, 2008). The resultant weak C—H···F—C interactions (Howard *et al.*, 1996; Reichenbacher *et al.*, 2005) arise mainly due to electrostatic and dispersive forces between the Cδ±-Fδ- and the Hδ±-Cδ- fragments. These interactions, at the borderline of the hydrogen bond phenomenon, are also poorly directional and are deformed by other dominant interactions (Weiss *et al.*, 1997; D'Oria & Novoa 2008; Müller *et al.*, 2007). In the absence of other interactions these weak interactions can play a role in the overall crystal packing of the molecule (Bats *et al.*, 2000; Choudhury *et al.*, 2004; Althoff *et al.*, 2006). In activated systems such as polyfluorobenzenes, C—H···F—C interactions may be of significance, and some of us had reported the crystal structures of several polyfluorinated benzenes in this connection (Thalladi *et al.*, 1998). As a continuation of this work, we report here the crystal structure of 1,3-difluorobenzene. The comparison crystal structures of 1,2- and 1,4-difluorobenzene and 1,3,5-trifluorobenzene have been reported in this earlier work.

### Experimental

Single crystals of 1,3-difluorobenzene were grown from commerical samples by zone melting in a quartz capillary at 163 K according to the procedure outlined by Boese & Nussbaumer (1994).

### Refinement

H atoms were positioned geoemtrically (C-H = 0.95 or 0.96 Å) and refined using a riding model, with their isotropic displacement parameters set equal to 1.2 times *U*^eq^ of the corresponding carbon atom.

### Crystal data

**Table:**

C_6_H_4_F_2_	*F*(000) = 928
*M**_r_* = 114.09	*D*_x_ = 1.445 Mg m^−^^3^
Monoclinic, *C*2/*c*	Mo *K*α radiation, λ = 0.71073 Å
Hall symbol: -C 2yc	Cell parameters from 2977 reflections
*a* = 24.6618 (13) Å	θ = 2.9–28.2°
*b* = 12.2849 (5) Å	µ = 0.13 mm^−^^1^
*c* = 7.2336 (4) Å	*T* = 153 K
β = 106.842 (3)°	Cylindric, colourless
*V* = 2097.55 (18) Å^3^	0.30 × 0.30 × 0.30 mm
*Z* = 16	

### Data collection

**Table:**

Bruker SMART APEXII area-detector diffractometer	2099 independent reflections
Radiation source: fine-focus sealed tube	1578 reflections with *I* > 2σ(*I*)
graphite	*R*_int_ = 0.020
Detector resolution: 512 pixels mm^-1^	θ_max_ = 28.3°, θ_min_ = 1.9°
Data collection strategy APEX 2/COSMO with chi +/– 10° scans	*h* = −27→29
Absorption correction: multi-scan (*SADABS*; Bruker, 2004)	*k* = −16→16
*T*_min_ = 0.876, *T*_max_ = 0.961	*l* = −9→8
7831 measured reflections	

### Refinement

**Table:**

Refinement on *F*^2^	Secondary atom site location: difference Fourier map
Least-squares matrix: full	Hydrogen site location: inferred from neighbouring sites
*R*[*F*^2^ > 2σ(*F*^2^)] = 0.032	H-atom parameters not refined
*wR*(*F*^2^) = 0.100	*w* = 1/[s^2^(*F*_o_^2^) + (0.0494*P*)^2^ + 0.6228*P*] where *P* = (*F*_o_^2^ + 2*F*_c_^2^)/3
*S* = 1.01	(Δ/σ)_max_ = 0.001
2099 reflections	Δρ_max_ = 0.19 e Å^−^^3^
146 parameters	Δρ_min_ = −0.13 e Å^−^^3^
0 restraints	Extinction correction: *SHELXTL* (Bruker, 2008), Fc^*^=kFc[1+0.001xFc^2^λ^3^sin(2θ)]^-1/4^
Primary atom site location: structure-invariant direct methods	Extinction coefficient: 0.0034 (7)

### Special details

**Table:**

Geometry. All e.s.d.'s (except the e.s.d. in the dihedral angle between two l.s. planes) are estimated using the full covariance matrix. The cell e.s.d.'s are taken into account individually in the estimation of e.s.d.'s in distances, angles and torsion angles; correlations between e.s.d.'s in cell parameters are only used when they are defined by crystal symmetry. An approximate (isotropic) treatment of cell e.s.d.'s is used for estimating e.s.d.'s involving l.s. planes.
Refinement. Refinement of *F*^2^ against ALL reflections. The weighted *R*-factor *wR* and goodness of fit *S* are based on *F*^2^, conventional *R*-factors *R* are based on *F*, with *F* set to zero for negative *F*^2^. The threshold expression of *F*^2^ > σ(*F*^2^) is used only for calculating *R*-factors(gt) *etc*. and is not relevant to the choice of reflections for refinement. *R*-factors based on *F*^2^ are statistically about twice as large as those based on *F*, and *R*- factors based on ALL data will be even larger.

### Fractional atomic coordinates and isotropic or equivalent isotropic displacement parameters (Å^2^)

**Table:**

	*x*	*y*	*z*	*U*_iso_*/*U*_eq_	
F1	0.20034 (3)	0.21738 (6)	0.63089 (12)	0.0520 (3)	
F2	0.01617 (3)	0.35397 (7)	0.49907 (14)	0.0587 (3)	
C1	0.16251 (5)	0.29864 (9)	0.56491 (16)	0.0349 (3)	
C2	0.10797 (6)	0.28273 (9)	0.56876 (17)	0.0372 (3)	
H2	0.0966	0.2170	0.6188	0.045*	
C3	0.07043 (6)	0.36645 (10)	0.49742 (18)	0.0370 (3)	
C4	0.08585 (6)	0.46202 (9)	0.42616 (17)	0.0378 (3)	
H4	0.0586	0.5187	0.3775	0.045*	
C5	0.14156 (6)	0.47408 (9)	0.42727 (16)	0.0370 (3)	
H5	0.1531	0.5394	0.3793	0.044*	
C6	0.18102 (6)	0.39238 (9)	0.49720 (17)	0.0355 (3)	
H6	0.2199	0.4008	0.4989	0.043*	
F11	0.23615 (3)	0.09707 (6)	0.01979 (13)	0.0571 (3)	
F12	0.05258 (4)	−0.03682 (6)	−0.08892 (13)	0.0625 (3)	
C11	0.18189 (6)	0.11273 (10)	0.01807 (17)	0.0365 (3)	
C12	0.14440 (6)	0.02770 (9)	−0.03976 (17)	0.0382 (3)	
H12	0.1558	−0.0409	−0.0800	0.046*	
C13	0.08994 (6)	0.04613 (9)	−0.03803 (18)	0.0385 (3)	
C14	0.07131 (6)	0.14411 (9)	0.01511 (18)	0.0380 (3)	
H14	0.0325	0.1545	0.0122	0.046*	
C15	0.11075 (6)	0.22716 (9)	0.07194 (17)	0.0383 (3)	
H15	0.0992	0.2961	0.1105	0.046*	
C16	0.16634 (6)	0.21260 (9)	0.07456 (18)	0.0392 (3)	
H16	0.1934	0.2705	0.1133	0.047*	

### Atomic displacement parameters (Å^2^)

**Table:**

	*U*^11^	*U*^22^	*U*^33^	*U*^12^	*U*^13^	*U*^23^
F1	0.0441 (6)	0.0416 (4)	0.0679 (5)	0.0105 (3)	0.0124 (4)	0.0059 (3)
F2	0.0295 (6)	0.0668 (5)	0.0841 (6)	−0.0076 (4)	0.0234 (5)	−0.0023 (4)
C1	0.0343 (9)	0.0338 (5)	0.0352 (6)	0.0013 (5)	0.0078 (6)	−0.0017 (4)
C2	0.0397 (9)	0.0341 (6)	0.0401 (6)	−0.0071 (5)	0.0153 (6)	−0.0012 (4)
C3	0.0257 (9)	0.0455 (6)	0.0411 (6)	−0.0062 (5)	0.0118 (6)	−0.0064 (5)
C4	0.0346 (9)	0.0382 (6)	0.0379 (6)	0.0027 (5)	0.0064 (6)	0.0002 (5)
C5	0.0405 (9)	0.0351 (6)	0.0362 (6)	−0.0046 (5)	0.0123 (6)	0.0017 (4)
C6	0.0267 (9)	0.0421 (6)	0.0395 (6)	−0.0053 (5)	0.0122 (6)	−0.0038 (5)
F11	0.0289 (6)	0.0654 (5)	0.0794 (6)	0.0116 (4)	0.0195 (5)	0.0076 (4)
F12	0.0502 (6)	0.0499 (5)	0.0894 (6)	−0.0173 (4)	0.0235 (5)	−0.0145 (4)
C11	0.0242 (9)	0.0468 (6)	0.0392 (6)	0.0077 (5)	0.0100 (6)	0.0073 (5)
C12	0.0425 (9)	0.0346 (6)	0.0402 (6)	0.0064 (5)	0.0161 (6)	0.0016 (5)
C13	0.0370 (9)	0.0375 (6)	0.0416 (6)	−0.0045 (5)	0.0122 (6)	−0.0014 (5)
C14	0.0283 (9)	0.0452 (6)	0.0433 (7)	0.0049 (5)	0.0149 (6)	0.0019 (5)
C15	0.0403 (9)	0.0357 (6)	0.0409 (6)	0.0049 (5)	0.0150 (6)	−0.0018 (4)
C16	0.0355 (9)	0.0381 (6)	0.0423 (7)	−0.0041 (5)	0.0086 (6)	−0.0017 (5)

### Geometric parameters (Å, °)

**Table:**

F1—C1	1.3553 (13)	F11—C11	1.3486 (14)
F2—C3	1.3506 (15)	F12—C13	1.3515 (14)
C1—C2	1.3673 (18)	C11—C12	1.3773 (17)
C1—C6	1.3797 (15)	C11—C16	1.3820 (16)
C2—C3	1.3800 (17)	C12—C13	1.3656 (18)
C2—H2	0.96	C12—H12	0.96
C3—C4	1.3793 (16)	C13—C14	1.3821 (16)
C4—C5	1.3794 (18)	C14—C15	1.3872 (17)
C4—H4	0.96	C14—H14	0.96
C5—C6	1.3874 (17)	C15—C16	1.3772 (18)
C5—H5	0.95	C15—H15	0.96
C6—H6	0.96	C16—H16	0.96
			
F1—C1—C2	117.92 (10)	F11—C11—C12	118.14 (11)
F1—C1—C6	118.36 (11)	F11—C11—C16	118.92 (11)
C2—C1—C6	123.71 (11)	C12—C11—C16	122.94 (12)
C1—C2—C3	116.30 (10)	C13—C12—C11	116.54 (11)
C1—C2—H2	121.7	C13—C12—H12	121.6
C3—C2—H2	122.0	C11—C12—H12	121.8
F2—C3—C2	118.12 (10)	F12—C13—C12	117.85 (10)
F2—C3—C4	118.76 (12)	F12—C13—C14	118.50 (11)
C2—C3—C4	123.12 (12)	C12—C13—C14	123.65 (11)
C3—C4—C5	118.11 (11)	C13—C14—C15	117.50 (12)
C3—C4—H4	121.0	C13—C14—H14	121.3
C5—C4—H4	120.9	C15—C14—H14	121.2
C4—C5—C6	121.10 (11)	C16—C15—C14	121.22 (11)
C4—C5—H5	119.5	C16—C15—H15	119.3
C6—C5—H5	119.4	C14—C15—H15	119.5
C1—C6—C5	117.64 (12)	C15—C16—C11	118.14 (11)
C1—C6—H6	121.2	C15—C16—H16	120.8
C5—C6—H6	121.2	C11—C16—H16	121.0

### Hydrogen-bond geometry (Å, °)

**Table:**

*D*—H···*A*	*D*—H	H···*A*	*D*···*A*	*D*—H···*A*
C2—H2···F12^i^	0.96	2.72	3.3750 (14)	126
C4—H4···F2^ii^	0.96	2.76	3.5386 (16)	139
C5—H5···F11^iii^	0.95	2.71	3.2948 (16)	121
C6—H6···F11^iii^	0.96	2.66	3.2644 (15)	121
C6—H6···F1^iv^	0.96	2.82	3.5789 (17)	137
C12—H12···F1^v^	0.96	2.70	3.3919 (14)	130
C14—H14···F2^vi^	0.96	2.72	3.3442 (16)	123
C14—H14···F12^vii^	0.96	2.73	3.5075 (18)	138
C15—H15···F2^vi^	0.96	2.81	3.3995 (17)	120
C16—H16···F11^viii^	0.96	2.75	3.5591 (16)	142
C2—H2···Cg2^ix^	0.96	2.96	3.6653 (13)	131
C12—H12···Cg2^v^	0.96	2.99	3.6547 (13)	127
C5—H5···Cg1^x^	0.95	2.83	3.5153 (12)	130
C15—H15···Cg1	0.96	2.87	3.5283 (13)	127

Symmetry codes: (i) *x*, −*y*, *z*+1/2; (ii) −*x*, −*y*+1, −*z*+1; (iii) −*x*+1/2, *y*+1/2, −*z*+1/2; (iv) −*x*+1/2, −*y*+1/2, −*z*+1; (v) *x*, −*y*, *z*−1/2; (vi) −*x*, *y*, −*z*+1/2; (vii) −*x*, −*y*, −*z*; (viii) −*x*+1/2, −*y*+1/2, −*z*; (ix) *x*, *y*, *z*+1; (x) *x*, −*y*+1, *z*−1/2.
